# Breast metastasis from gastric carcinoma: A case report

**DOI:** 10.22088/cjim.13.1.132

**Published:** 2022

**Authors:** Leila Kiani Markani, Fatemeh Kiani Markani, Maryam Kadivar, Negar Hossinaei, Elahe Safari

**Affiliations:** 1 Department of Pathology, Rasool Akram Medical Complex, Iran University of Medical Sciences, Tehran, Iran; 2 Department of Immunology, School of Medicine, Iran University of Medical Sciences, Tehran, Iran

**Keywords:** Breast, gastric carcinoma, metastasis

## Abstract

**Background:**

There are rare cases of breast metastasis from gastric carcinoma origin. In this regard, we presented a case of signet ring cell stomach cancer with metastasis to the breast.

**Case Presentation:**

The case was a 44-year-old female with a history of gastric cancer and chief complaint of progressive bilateral and gradual breast enlargement and mass palpation 6 years after stomach surgery. An excisional biopsy of the right breast was performed at the early phase of her clinical symptoms with the pathology at the early phase of fibroadenoma and sclerosing adenosis. Given the persistent right breast thickening and enlargement, the ultrasonography and MRI together showed right breast large masses, ductal enhancement in left retro areolar space beside the bilaterally enlarged axillary lymph nodes after 6 months. In this phase, the core needle biopsy showed right breast mass adenocarcinoma consistently with metastatic gastric carcinoma and also core needle biopsy of both axillary lymph nodes indicating the involvement by tumor consistently with metastatic gastric carcinoma. In the IHC, breast tumor cells were negative for ER, PR, HER2/ neu, GATA3, GCDFP15 and CK20, but positive for CK7, CK19, and CDx2.

**Conclusion:**

The diagnosis of breast metastasis of gastric carcinoma was confirmed according to the past history of patient, histological finding, and immune-histological markers.

Gastric cancer ranked second among the other cancers with a higher incidence in Asia more than North America and Central and Eastern Europe. In Asian countries, patients are diagnosed in earlier stages than other countries (1). A total of 720,000 patients passed away from gastric cancer in 2012 with more than 950,000 new cases per year worldwide (2) causing 23% of lung and 28% of liver cancers (3). Adenocarcinoma, which accounts for 90% of gastric cancers, is further divided into diffuse and intestinal according to the Lauren criteria. Intestinal cells are adhesive and arranged in glandular or tubular forms. Intestinal metaplasia is usually associated with this type. In the diffuse type, despite intestinal, tumor cells are scattered and lack any adhesion. Intracellular mucus may push the cell nucleus aside to form signet-ring cell carcinoma. The diffuse type has a worse prognosis than the intestinal type (4, 5). Intra-abdominal recurrence is a significant feature of gastric carcinoma with the most common site liver, peritoneal membrane, but the metastasis of breast is hardly seen. Not only the prognosis is poor in this patient, but also the exact mechanism which causes gastric cancer metastases to the breast is unknown yet (6, 7). The highest possibility includes 2 routs: lymphatic or blood vessel (6). 

## Case Presentation

In March 2011, a 36-year-old female had severe epigastria pain, hematemesis, nausea and vomiting.

She was admitted to Loghman Hospital of Tehran, Iran, and the diagnosis of gastric cancer confirmed by endoscopy. A partial gastrectomy was done to her. Tumor from antral part of stomach was sent to pathology department. Tumor size was 4.5-3.5 cm in area and 1 cm in maximum thickness. The section had a neoplasm composing of atypical epithelial cells with hyperchromatic nuclei, rather deeply stained cytoplasm and times vacculated and signet ring type. They were separately arranged in Indian filled pattern infiltrating in tantacular fashion throughout the entire thickness of gastric wall that reached to serosol layer and perineural. On the other hand, 8 out of 17 respected lymph nodes indicated the metastatic involvement, but no blood vessel involvement. IHC staining was positive for CK7, but negative for LCA, NSE, and chromogranin. The diagnosis was adenocarcinoma, infiltrative and poorly differentiated and the chemotherapy and radiotherapy were done for the patient.

It should be noted that she had two pregnancies in 10-11 months. She generally had difficult pregnancies and did not eat well during the pregnancy. Even she vomited blood. Severe intolerable stomach ache was mistakenly diagnosed as a kidney disease during the breast-feeding period and she started kidney therapy. After 4 months (8 months from disease initiation), she was referred to gastroscopy because of nausea, vomiting and diarrhea, and stomach cancer was diagnosed. 

In December 2016, the patient was transferred to Mehrad Hospital with the start of intense pain in lower abdomen and 40-50 intervals between periods and also chief complaints of progressive bilateral and gradual breast enlargement and mass palpation. Left salpingo-oophorectomy had been done and the specimen was sent for further examination to the pathology department. The specimen consisted of a fresh encapsulated creamy solid echogenic oval shape mass that was attached to the fallopian tube with 7*5.5*5 cm of diameters and 93g of weight. Result was negative for malignancy, but the section had a neoplasm composing of foscicles of spindle cells with centrally-placed nuclei and moderate pale cytoplasm. The cellularity was low and the mitotic activity was rare. IHC staining was positive for inhibin, but negative for CK, SMA, vimentin Ki67 <1%. Furthermore, the right breast excisional biopsy was performed, and the hypoechoic mass was 7*6*5 cm in diameter and 85 g in weight (B4). Cut section revealed gray-creamy surface with firm consistency; and the mass was negative for malignancy, but it had a fibroadenoma pericanalicular type indicating adenosis and cystic apocrine metaplasia.

In June 2017, the sonography of both breasts and axillary regions was performed by 5 MHZ to 18 MHZ of multifrequency probe. The breast parenchyma pattern was heterogeneous fibroglandular. Multiple hypoechoic masses (breast composition C) was seen in both breasts. The most prominent ones were as follows: right breast mass: 66*20 mm hypoechoic structure at UOQ mid-zone compatible with the enhancing region on MRI (B4b) (figure); and 24*15 mm similar hypoechoic structure at a medial retroaleolar part. Core needle biopsy was done from right breast mass in formalin consisting of 5 cores of gray-creamy, soft to rubbery tissue with 0.5 cm to 1.8 cm of length and 0.2 cm of the greatest diameter. The adenocarcinoma was diagnosed with metastatic gastric carcinoma, fibrocystic changes with foci of sclerosing adenosine with microcalcification including ossifying type. IHC staining was negative for ER, PR, HER2/neu, GATA3, GCDFP15, and CK20, but positive for CDX2, CK19, CK7, Pan-CK, E-Cadherin, and CEA. Sonography of the left breast was skin thickening at the medial areolar part up to 6.5 mm that was compatible with the observed enhancement in MRI. There was ductal enhancement in the left retroareolar part. Sonography of axillary regions revealed prominent lymph nodes in both sides with cortical thickening. One of them at the right axillary region indicated the focal eccentric cortical thickening and had an indeterminate appearance. The core needle biopsy was done for right axillary lymph nodes. It consisted of 3 cores of gray creamy, soft to rubbery tissues with 1.5 cm to 2.3 cm of length and 0.2 cm of the greatest diameter. Pathological evaluation of the lymphoid tissue was infiltrated by metastatic adenocarcinoma that was positive for CK7 and some of them positive for CDX2 in IHC staining; hence, the metastatic left axillary lymph node was diagnosed with the most probable gastric origin. 

Similarly, the axial spiral CT-scan of brain with the contrast media indicated that there was no evidence of mass or abnormality in the brain. The ventricular system was intact; and the posterior fossa and ventricular were normal; the axial spiral CT of chest showed a star shape fibrosis in apical segment of the left upper lung, but no evidence of pulmonary metastasis was detected. Broncho vascular models of both lungs were normal. CT of abdominopelvic cavity with contrast showed fatty changes in both liver lobes; the spleen was normal in size and density; kidneys symmetrically excreted contrast without any evidence of hydronephrosis or mass; and there was no significant retroperitoneal adenopathy. The 4*3 cm functional ovarian cyst was seen in the right adnexal area. The whole-body scan was done 2hrs after the injection IV of 740MBq^99m ^TC MDP. The multiple bone metastases were detected in the sternum, both clavicles, right shoulder, spines at T4, T5, T9, S1 joints, sacrum, right iliac crest and probably in upper cervical vertebrae and right greater trochanter. MRI of the total spine was done with and without the contrast media. Foci of T1 and T2 prolongation with post-contrast enhancement were seen at C2, C5, T5, T9, T 11 and L2 highly suggestive of osteolytic metastasis. Disc bulge was seen at L4-5 with a posterior annular tear, but without any herniation. Chemotherapy was done for the patient, but she succumbed to the disease after 15 months.

**Figure 1:**
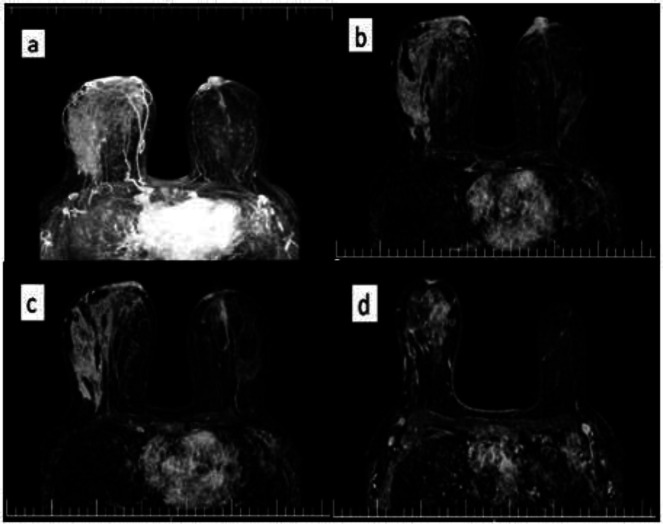
MRI of breasts and both axillae with pre contrast axial T1 and T2w image technique. a. MRI / MIP subtraction images: extensive enhancement of right breast in lateral and anterior portion + enhancement and enlargement of left nipple along with areolar skin enhancement linear non mass enhancement at central retro- nipple. b, c & d: MRI subtraction images significant regional / multiple regions of enhancement in right breast and significant enlargement + enhancement of left nipple associated with thickening and focal enhancement of areolar skin

## Discussion

The metastatic breast cancer from the extramammary gland is merely diagnosed at 1.7%-6.6% based on autopsy studies of patients with any type of cancer, 1.2% in clinical reports and 2.7 in cytological review (8). Breast metastasis is hardly seen in the gastric carcinoma. In 1999, the first case was reported with metastasis in breast and ovary from gastric signet ring cell (9). There are 63 patients of whom 62 cases were women worldwide. The metastasis to the breast was not only seen in these cases, but also the most other sites were ovary and axillary lymph nodes. According to the available data, 36 cases had signet ring cells and 8 adenocarcinomas (7), but, cases of the present study had both signet ring cells and adenocarcinoma.

There was not any report in this regard in Iran; hence, it was the first report. When the patient was diagnosed with gastric carcinoma, she just had 8 out of 17 lymph nodes and perineural invasion. After 6 years, she came back with the left adnexal and right breast mass with no malignancy; however, the right breast and bones showed metastasis from gastric carcinoma after 6 months. According to results, only 2 of them had bone metastasis. The mean age of a diagnosed patient with metastasis to the breast was 43 (7). Our case had the breast metastasis at 43 years of age. In China, the shortest interval to the breast metastasis was 40 days and the longest was 5 years, despite our patient with the interval of 6 years. 

In general, the metastatic masses seem benign without any sign of microcalcification in the mammography. These masses are mainly found in the upper outer quadrant of the leafy breast that is palpable. Both breasts' involvement was seen in 25% of cases. Up to 15% of those patients had axillary lymph node metastasis (10, 11), but our patient had metastasis in the right breast, and like some cases, she had axillary lymph node metastasis, but unlike others, she had microcalcification in the right breast.

Immunohistochemistry techniques can be helpful for distinguishing the primary origin of tumor in metastatic cancer. Tumor markers, CEA, CK7, and CK20 are positive and negative for estrogen receptor (ER) and progesterone receptor (PR) in metastatic gastrointestinal tumors. Therefore, we can use a combined staining of CK20 and CEA positive/negative ER and PR for an accurate diagnosis. This is exactly as same as our patient who had ER and PR positive tumor and CK7 and CK20 positive (12-14). Breast cancers, which are triple negative (like our case), tend to have the least favorable survival rates regardless of the breast cancer stage.

 In conclusion, Breast metastasis, which has a gastric origin, had more than 80 probabilities of death during a year. Histopathological techniques such as immunohistochemistry for tumor markers CK20, CK7, CDX2 were helpful to diagnose the origin of cancer (15); and the better therapeutic management could cause a better survival rate.

## Conflict of Interests:

There are no conflicts of interest.

## References

[1] Okines A, Verheij M, Allum W (2010). Gastric cancer: ESMO Clinical Practice Guidelines for diagnosis, treatment and follow-up. Ann Oncol.

[2] Ferlay J, Steliarova-Foucher E, Lortet-Tieulent J (2013). Cancer incidence and mortality patterns in Europe: estimates for 40 countries in 2012. Eur J Cancer.

[3] Soerjomataram I, Lortet-Tieulent J, Parkin DM (2012). Global burden of cancer in 2008: a systematic analysis of disability-adjusted life-years in 12 world regions. Lancet.

[4] Ma J, Shen H, Kapesa L, Zeng S (2016). Lauren classification and individualized chemotherapy in gastric cancer. Oncol Lett.

[5] Chen YC, Fang WL, Wang RF (2016). Clinicopathological variation of Lauren classification in gastric cancer. Pathol Oncol Res.

[6] Wei LY, Kong M, Zhang Z, Zhang XC (2017). Breast metastasis of gastric signet-ring cell carcinoma. J Zhejiang Univ Sci B.

[7] Dulskas A, Al Bandar M, Choi YY (2017). A case of gastric cancer metastasis to the breast in a female with BRCA2 germline mutation and literature review. Acta Chir Belg.

[8] Tian Q, Zeng J, Tao X (2016). Clinical pathology of metastatic gastric carcinoma to the breast: A report of two cases and a review of literature. Oncol Lett.

[9] Kwak JY, Kim EK, Oh KK (2000). Radiologic findings of metastatic signet ring cell carcinoma to the breast from stomach. Yonsei Med J.

[10] Yan H, Liu J, Ming X (2017). Metastatic gastric carcinoma to the breast: A case report and review of the Chinese literature. Mol Clin Oncol.

[11] Qureshi SS, Shrikhande SV, Tanuja S, Shukla PJ (2005). Breast metastases of gastric signet ring cell carcinoma: a differential diagnosis with primary breast signet ring cell carcinoma. J Postgrad Med.

[12] Madan AK, Ternovits C, Huber SA, Pei LA, Jaffe BM (2002). Gastrointestinal metastasis to the breast. Surgery.

[13] Boutis AL, Andreadis C, Patakiouta F, Mouratidou D (2006). Gastric signet-ring adenocarcinoma presenting with breast metastasis. World J Gastroenterol.

[14] Briest S, Horn LC, Haupt R (1999). Metastasizing signet ring cell carcinoma of the stomach—mimicking bilateral inflammatory breast cancer. Gynecol Oncol.

